# Function of COP9 Signalosome in Regulation of Mouse Oocytes Meiosis by Regulating MPF Activity and Securing Degradation

**DOI:** 10.1371/journal.pone.0025870

**Published:** 2011-10-03

**Authors:** Eunju Kim, Se-Jin Yoon, Eun-Young Kim, Yunna Kim, Hyun-Seo Lee, Kyeoung-Hwa Kim, Kyung-Ah Lee

**Affiliations:** 1 Department of Biomedical Science, College of Life Science, CHA University, Seoul, Korea; 2 Department of Genetics, Stanford University School of Medicine, Stanford, California, United States of America; 3 CHA Research Institute, Fertility Center, CHA General Hospital, Seoul, Korea; St. Georges University of London, United Kingdom

## Abstract

The COP9 (constitutive photomorphogenic) signalosome (CSN), composed of eight subunits, is a highly conserved protein complex that regulates processes such as cell cycle progression and kinase signalling. Previously, we found the expression of the COP9 constitutive photomorphogenic homolog subunit 3 *(CSN3)* and subunit 5 (*CSN5*) changes as oocytes mature for the first time, and there is no report regarding roles of COP9 in the mammalian oocytes. Therefore, in the present study, we examined the effects of RNA interference (RNAi)-mediated transient knockdown of each subunit on the meiotic cell cycle in mice oocytes. Following knockdown of either *CSN3* or *CSN5*, oocytes failed to complete meiosis I. These arrested oocytes exhibited a disrupted meiotic spindle and misarranged chromosomes. Moreover, down-regulation of each subunit disrupted the activity of maturation-promoting factor (MPF) and concurrently reduced degradation of the anaphase-promoting complex/cyclosome (APC/C) substrates Cyclin B1 and Securin. Our data suggest that the *CSN3* and *CSN5* are involved in oocyte meiosis by regulating degradation of Cyclin B1 and Securin via APC/C.

## Introduction

The COP9 signalosome (CSN) was first identified in *Arabidopsis thaliana* as a repressor of photomorphogenesis and is conserved evolutionally from yeast to human [1], [2]. This complex is composed of eight independent subunits, *CSN1* to *CSN8* [1], [2], and has subunit-to-subunit similarity with the 26S proteasome lid complex [3], but has different functions. The CSN interacts with several kinases [4], [5], [6], recruits deubiquitination enzymes, and has an intrinsic isopeptidase activity for deneddylation [7], [8]. With these activities, the CSN is involved in regulating various processes including invertebrate development [9], cell cycle [10], [11], [12], kinase signaling [13], and nuclear transport [14]. Moreover, it regulates protein subcellular localization [15], [16], [17] and transcriptional activities [18].

Several developmental defects arise following loss of some CSN subunits. Mutations in the CSN complex in *Arabidopsis* resulted in delayed G2 progression [19]. Defects in oogenesis and embryo patterning, as well as larval lethality, occurred in *Drosophila melanogaster* with an impaired CSN complex [20], [21]. Likewise, reducing CSN function by RNAi in *Caenorhabitis elegans* resulted in sterility and alterations in microtubule structure [22]. In mice, disruption of *CSN2* or *CSN3* led to impaired cell proliferation, accumulation of p53 and Cyclin E, and early embryonic death [23], [24]. *CSN5*-deficient mouse ES cells also displayed dysfunctional proliferation and accelerated apoptosis [20]. Although some reports indicate an essential role of the CSN in mammalian early embryonic development and cell cycle regulation, the function of CSN subunits in mammalian oocyte meiotic maturation remains poorly understood.

Previously, we reported the expression of the *CSN3* and *CSN5* subunits in mouse oocytes [25]. *CSN3* binds to CSN associated kinases [5], and *CSN5* (also known as *Jab1*) can associate with signaling molecules such as p53 [26], p27 [27], and c-Jun [18]. In this study, we investigated the effects of *CSN3* or *CSN5* loss-of-function on oocyte meiotic cell cycle. Because of the lethality caused by CSN-deficiency [24], [28], oocyte-targeting genetic manipulation rather than generation of knockout mutants is required to evaluate the potential role of these subunits in oocytes. Therefore, knockdown of specific subunits by RNA interference (RNAi), which ablates target gene expression spatially and temporally, was performed in this study. Our results demonstrate that *CSN3* or *CSN5* knockdown leads to meiosis I arrest, disruption of maturation promoting factor (MPF) activity, and decreased degradation of anaphase-promoting complex/cyclosome (APC/C) substrates.

## Materials and Methods

### Animals

All ICR mice were obtained from Koatech (Pyeoungtack, Korea) and maintained in the animal facility of the CHA Stem Cell Institute of CHA University. All procedures described within were reviewed and approved by the University Institutional Animal Care and Use Committee, and performed in accordance with the Guiding Principles for the Care and Use of Laboratory Animals.

### Collection of oocytes and follicular cells

Three-week-old female mice were injected intraperitoneally with 5 IU eCG (Sigma-Aldrich, St. Louis, MO, USA) and were sacrificed 46 h later. Isolated ovaries were punctured in M2 medium (Sigma-Aldrich) and cumulus-oocyte complexes (COCs) were collected. Cumulus cells (CCs) were mechanically detached from COCs, and the denuded oocytes were collected for microinjection and culture. CCs and granulosa cells (GCs) were frozen after washing with PBS.

### Preparation of dsRNA, microinjection, and *in vitro* oocyte culture

A portion of the *CSN3* or *CSN*5 mRNA sequence was cloned into the pGEM-T easy vector (Promega, Madison, WI, USA). After linearization with SpeI, each RNA strands were transcribed *in vitro*, annealed at 75°C, and then cooled to room temperature. The long double-stranded RNA (dsRNA) was purified with DNaseI and RNase digestion prior to dilution to 2.3 µg/µl. Germinal vesicle (GV) oocytes were microinjected with the dsRNAs in M2 medium. To inhibit germinal vesicle breakdown (GVBD) during collection and microinjection, 0.2 mM isobutylmethylxanthine (IBMX; Sigma-Aldrich) was added to the M2 medium. An injection pipette containing dsRNA solution was inserted into the cytoplasm of an oocyte, and approximately 4.1×10^7^ dsRNA molecules in 10 pl was microinjected using a constant-flow system (Transjector; Eppendorf, Hamburg, Germany). For complete degradation of target transcripts before maturation, oocytes were cultured in IBMX-supplemented M16 medium for 8 h and then cultured for 16 h in M16 medium alone before scoring maturation rates in 5% CO_2_ at 37°C. When scoring maturation rates, oocytes with the first polar body were considered as MII, and oocytes without GV or any polar bodies were considered to be in the MI stage.

### Messenger RNA isolation and quantitative real-time RT-PCR

Oocyte mRNA was isolated using the Dynabeads mRNA DIRECT Kit (Dynal, Oslo, Norway) according to the manufacturer's instructions. Briefly, oocytes were suspended with lysis/binding buffer and mixed with prewashed Dynabeads oligo dT_25_. After RNA binding, the beads were washed with buffer A twice, followed by buffer B, and RNA was eluted with Tris-HCl by incubation at 70°C. The isolated mRNA was used as a template for reverse transcription using oligo (dT) primers according to the MMLV protocol. PCR was carried out with single-oocyte equivalent amount of cDNAs and primers (Table 1). For quantitation of mRNA, quantitative real-time RT-PCR was performed with single-oocyte equivalent amount of cDNA as previously described [29].

**Table 1:** Sequences of oligonucleotide primers and their expected RT-PCR product sizes.

Gene	Accession No.	Oligonucleotide Sequences^a^	AT (°C)	Product Size (bp)
*CSN3_A* b	NM_011991	F : CAGCTGCCTAAATACACCTCR : GAGTCATCTTCTTGTGAGCC	60	527
*CSN3_B* c	NM_011991	F : ATCGACCAAGAGATGCTAAAR : CTTTGCAGAGAATGGTTTTC	60	307
*CSN5_A* b	NM_013715	F : CTGTAGAGGGCACAGAAACTR : TCCGACTGCTCTAACTTCTC	60	535
*CSN5_B* c	NM_013715	F : AGGCAACTTGGAAGTGATGGR : TAACATCAATCCCGGAGAGC	60	242
*CSN1*	NM_145370	F : CAAGGCCGAGTCTACTCCAGR : CTCCAGGAACAGCTTGAAGG	60	295
*CSN2*	NM_009939	F : CAACAGTGCAGAGATGTGACR : ATAGACGGACACAGTTTTGG	60	459
*CSN4*	NM_012001	F : TCGGATGCTGGCTACTCTTTR : GGATCTGCTTGTCCCATGTT	60	395
*CSN6*	NM_012002	F : GAGTGTTTCCGTCGCTCTTCR : CAACCCAGAAACTCCAGCTC	60	261
*CSN7*	NM_012003	F : TCAGCGGCTAGAGGTTGATTR : TCTGTCAGGTGTTGCTCAGG	60	276
*CSN8*	NM_133805	F : TCTGTGGAAGAGGATACCACR : TGAAGGTGGATCTTGAACTC	60	497
*Gapdh*	BC092294	F : ACCACAGTCCATGCCATCACR : TCCACCACCCTGTTGCTGTA	60	451
*Gdf9*	NM_008110	F : GGTTCTATCTGATAGGCGAGGR : GGGGCTGAAGGAGGGAGG	60	446
*H1foo*	NM_138311	F : GCGAAACGGAAAGAGGTCAGAAR : TGGAGGAGGTCTTGGGAAGTAA	60	378
*Fshr*	NM_013523	F : TCCTTCATGGGACTGAGCTTR : AGAGGCTCCCTGCAAAACAT	60	165

a F, forward; R, reverse.

b Primers were used for preparation of dsRNA.

c Primers were used for confirmation the knockdown of target mRNA after RNAi.

### Visualization of spindle structures and chromosomes

The spindle structures of oocytes were noninvasively visualized using Pol-Scope (Oosight Meta Imaging System; CRI Inc., Woburn, MA, USA). To visualize chromosome arrangement, oocytes were fixed with methanol and acetic acid, stained with Aceto-Orcein on glass slides, and analyzed using the Cool-scope (Nikon Instruments Inc., NY, USA). For immunofluorescence staining, oocytes were fixed in PBS containing 0.1% polyvinyl alcohol (PVA-PBS), 4% paraformaldehyde, and 0.2% Triton X-100 for 40 min at room temperature. Fixed oocytes were washed with PBS-PVA, blocked with 3% BSA-PVA-PBS for 1 h and incubated with the mouse monoclonal anti-α-Tubulin antibody (1∶100 dilution; sc-8035; Santa Cruz Biotechnology) at 4°C overnight. And then, incubated with fluorescein isothiocyanate-conjugated anti-mouse IgG (1∶40; Sigma) for 1 h at room temperature, and DNA was counterstained with propidium iodide (Sigma).

### Western blot

Protein extract (100 oocytes/lane) was subjected to 12% SDS-PAGE and transferred onto a polyvinylidene difluoride (PVDF) membrane (Amersham Biosciences, Piscataway, NJ). The membrane was blocked in Tris-buffered saline-Tween (TBS-T) containing 5% skim milk and incubated with antibodies against CSN3 (1∶1000; sc-100693, Santa Cruz Biotechnology, Santa Cruz, CA, USA), CSN5 (1∶1000; sc-13157, Santa Cruz Biotechnology), Cdc2 (1∶1000; sc-54, Santa Cruz Biotechnology), Cyclin B1 (1∶1000; sc-245, Santa Cruz Biotechnology), Securin (1∶2000; DCS-280, Abcam, Cambridge, UK), β-actin (1∶2000;sc-8432, Santa Cruz Biotechnology), and α-tubulin (1∶2000; sc-8035, Santa Cruz Biotechnology) followed by incubation with horseradish peroxidase-conjugated anti-mouse immunoglobulin (1∶2000; A-2554, Sigma-Aldrich). After each step, the membranes were washed with TBS-T. Bound antibody was detected using Western blotting luminol reagents (Santa Cruz Biotechnology). Protein levels were quantified by measuring the density and area for each band using ImageJ software (NIH). These values were then normalized to α-tubulin, and were expressed as a percentage of control oocytes.

### Dual kinase activity assay

For measure the activity of the two kinases, MPF and MAPK, dual kinase activity assay was performed as previously described [29]. At each 2 h interval of *in vitro* culture, oocytes were collected, washed in 0.1% PVA-PBS, lysed with 4 µl of lysis buffer, and then frozen at -80°C until assayed. After thawing, oocytes were added of 5 µl kinase buffer containing 0.3 µCi/µl [γ-^32^P]-ATP (250 µCi/25 µl; Amersham Bioscience) and 5 µl substrates and incubated for 20 min at 37°C. The substrate solution for the MPF and MAPK double-kinase assay contained 4.5 µl histone H1 and 0.5 µl myelin basic protein (MBP). The reaction was terminate by the addition of 5 µl of 4× SDS sample buffer and boiling for 5 min. Samples were separated by 15% PAGE, and then labelled MBP and Histone H1 were analyzed by autoradiogram. Kinase activity was quantified by measuring the area of each lane using ImageJ (NIH). The values are presented as a ratio relative to control oocytes at 2 h.

### Statistical analysis

Each experiment was performed at least three independent times. The data were analyzed using the one-way ANOVA and presented as the mean ± SEM. A value of p<0.05 was considered to be statistically significant.

## Results

### Expression of *CSN3* and *CSN5* during oocyte maturation

**Figure 1:**
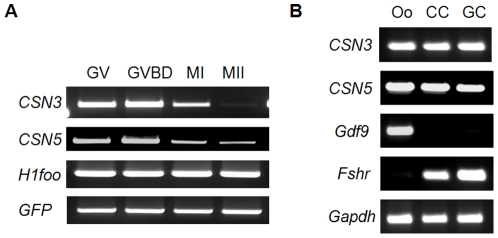
Expression of *CSN3* and *CSN5* mRNA in oocytes and follicular cells. (A) Expression of *CSN3* and *CSN5* mRNA in oocytes during in vitro maturation. For semi-quantitative PCR, single oocyte-equivalent cDNA was used as a template for amplification. GV, GVBD, MI, and MII oocytes were harvested at 0, 2, 8, and 16 h, respectively. Constitutively expressed oocyte-specific *H1foo* was used as an internal control and synthetic *GFP* mRNA was added to the lysates before mRNA isolation to compare the efficiency of mRNA recovery. (B) Expression of *CSN3* and *CSN5* mRNA in follicular components. Oo, oocytes at GV stage; CC, cumulus cells; GC, granulosa cells. *Gdf9* and *Fshr* were used as markers for oocytes and granulosa cells, respectively.

During *in vitro* oocyte maturation, *CSN3* and *CSN5* mRNA expression was highest in GV and GVBD oocytes and gradually decreased as oocytes mature (Fig. 1A). In ovarian follicles, CCs and GCs, as well as GV oocytes, expressed *CSN3* and *CSN5* (Fig. 1B). Expression of *Gdf9* and FSH receptor (*Fshr*) was used as markers for oocytes and GC cells, respectively.

### RNAi-mediated knockdown of *CSN3* and *CSN5* was specific

**Figure 2:**
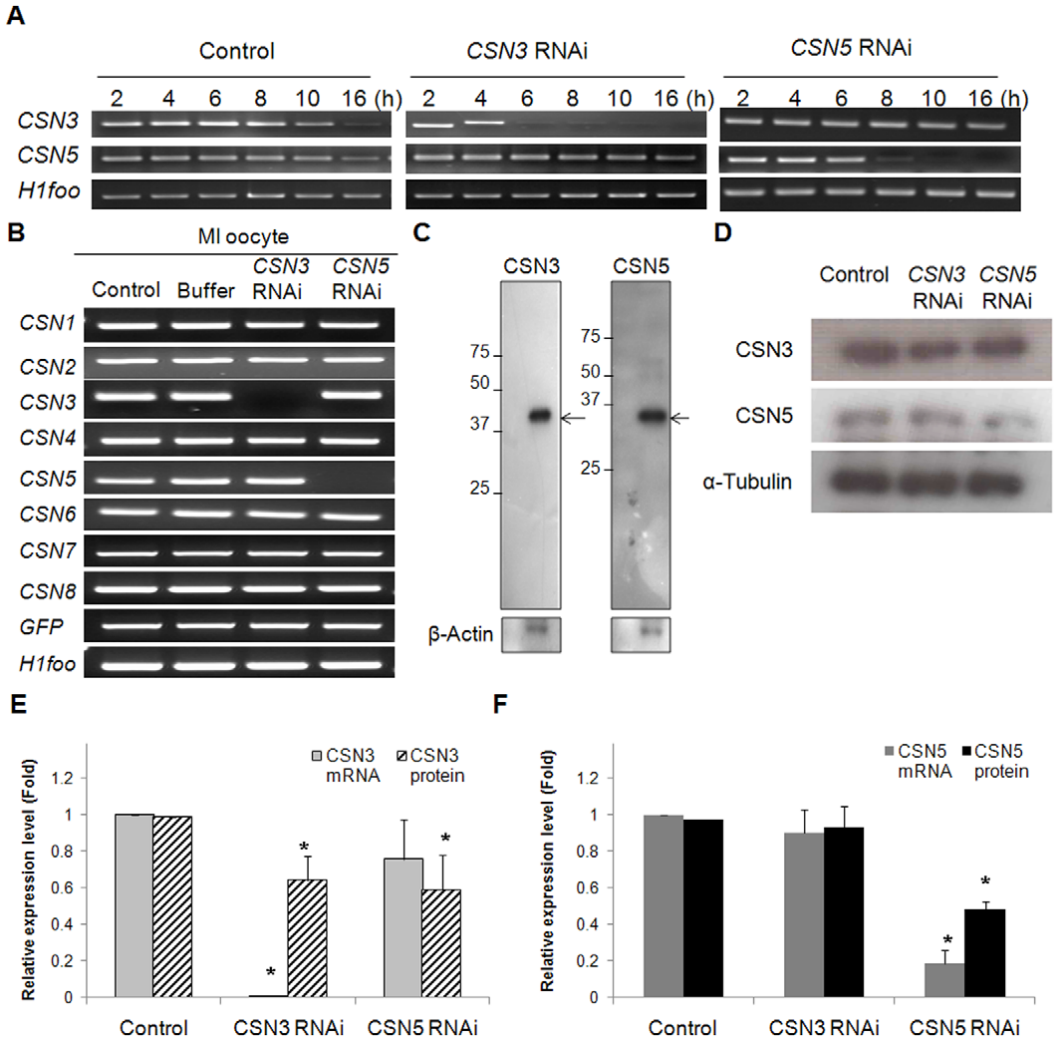
Efficiency of RNAi-mediated knockdown of *CSN3* and *CSN5*. (A) Determination of the critical time point for complete knockdown of *CSN3* and *CSN5* after RNAi. Oocytes were collected every 2 h after RNAi, and *CSN3* and *CSN5* mRNA was assessed by RT-PCR. (B) Specific suppression of *CSN3* or *CSN5* mRNA by RNAi. The other CSN subunits were not affected by RNAi treatment. *H1foo* was used as an internal control of oocytes, and exogenous *GFP* mRNA was used as an external control. (C) Western blot analysis of CSN3 and CSN5. The blot was incubated with each antibody using 100 oocytes. Numbers on the left side of the band indicate the sizes (kDa) of the protein markers, while arrows indicate the specific proteins. β-Actin was used as a loading control. (D) Protein knockdown after RNAi. Levels of CSN3 and CSN5 protein in RNAi-treated oocytes were determined using Western blot analysis. Proteins were extracted from 100 MI oocytes for each lane. (E, F) Bar graphs show the relative mRNA and protein levels after RNAi. The mRNA level was calculated with quantitative real-time RT-PCR using single-equivalent oocyte cDNA, while protein level was calculated by measuring the density and area of the bands. The mRNA and protein levels are presented relative to those of control oocytes. Asterisks indicate statistically significant differences compared to that of control or buffer group (p<0.05).

To determine rate of mRNA degradation after RNAi, oocytes were collected at every 2 h during *in vitro* maturation and target gene expression was assessed by RT-PCR (Fig. 2A). In control oocytes, *CSN3* and *CSN5* mRNA was gradually decreased during the culture of oocytes with IBMX. After RNAi, *CSN3* mRNA was completely abolished within 6–8 h but *CSN5* mRNA was around 4–6 h. RNAi was sequence-specific and expression of these mRNAs were not affected by each other's RNAi.

After *CSN3* RNAi, only *CSN3* mRNA was degraded and the other subunits were not affected (Fig. 2B). The same was true for *CSN5* RNAi. It indicates that depletion of specific subunit by RNAi was sequence-specific. To confirm the knockdown of the target protein, we performed Western blot analysis. A single, specific endogenous band of *CSN3* and *CSN5*, respectively, was detected in oocyte protein with the specific antibodies used (Fig. 2C). Results showed a marked decrease of CSN3 and CSN5 proteins by RNAi of each subunit (Fig. 2D) as expected. Microinjection of *CSN5*-targeted RNAi reduced CSN5 by 52% without altering CSN3 expression (Fig. 2E–F). However, unexpectedly, *CSN3*-targeted RNAi diminished CSN3 by 40% and concurrently CSN5 protein by 42%.

### Knockdown of *CSN3* or *CSN5* causes meiotic arrest at MI

**Figure 3:**
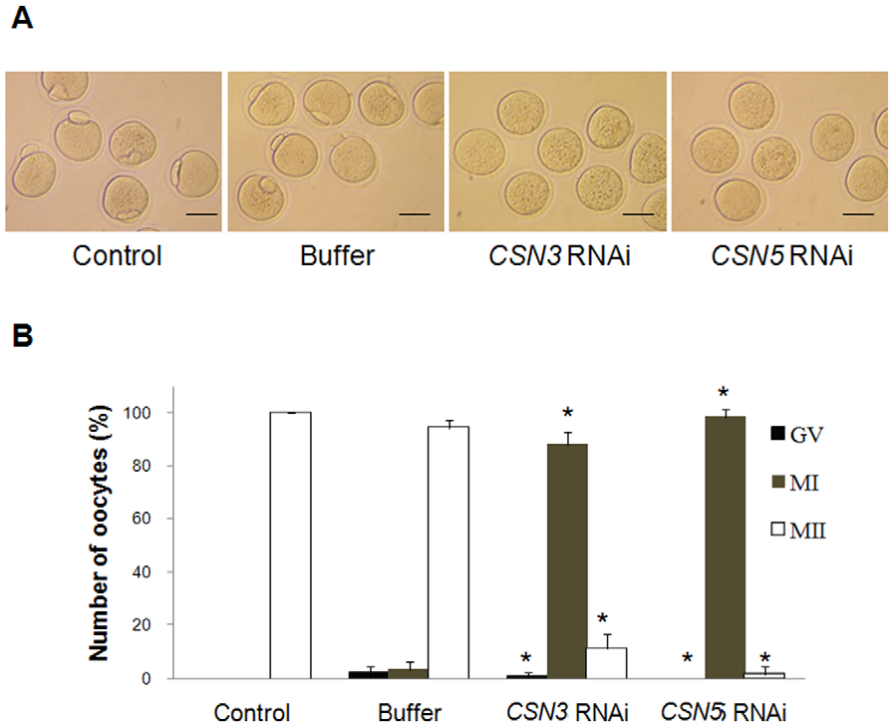
Microinjection of *CSN3* or *CSN5* dsRNA into GV oocytes resulted in MI arrest. Microphotographs of oocytes (A) and maturation rates (B) after *in vitro* culture for 16 h. Control and buffer-injected groups were MII after 16 h in culture, but injected oocytes were arrested at the MI stage. Bars = 50 µm. Asterisks indicate statistically significant differences compared to that of control or buffer group (p<0.05).

While control and buffer-injected mock control oocytes completed meiosis process and became MII oocytes with extrusion of the first polar body at the end of 16 h of culture, *CSN3* or *CSN5* RNAi-treated oocytes were arrested at the MI stage (Fig. 3A). In particular, 88% and 98.3% of *CSN3*- and *CSN5*-RNAi treated oocytes, respectively, were arrested at the MI stage (Fig. 3B, Table 2). No lysis was observed, but some oocytes showed lumpy ooplasmic membranes with a crumpled cytoplasm.

**Table 2:** *in vitro* maturation of mouse oocytes after *CSN3* or *CSN5* RNAi.

	No. of oocytes (%)				
Treatment	Total	GV	MI	MII	
Control	69	0 (0.0)	0 (0.0)	69	(100.0)
Buffer-injected	94	2 (2.3)	3 (3.5)	89	(94.2)
*CSN3* RNAi	127	1 (0.9)	112 (88.0)*	14	(11.2)*
*CSN5* RNAi	123	0 (0.0)	121 (98.3)*	2	(1.7)*

*Values are statistically significant at p<0.05.

### Disruption of meiotic spindle and chromosome structure in *CSN3*- or *CSN5*-RNAi treated oocytes

**Figure 4:**
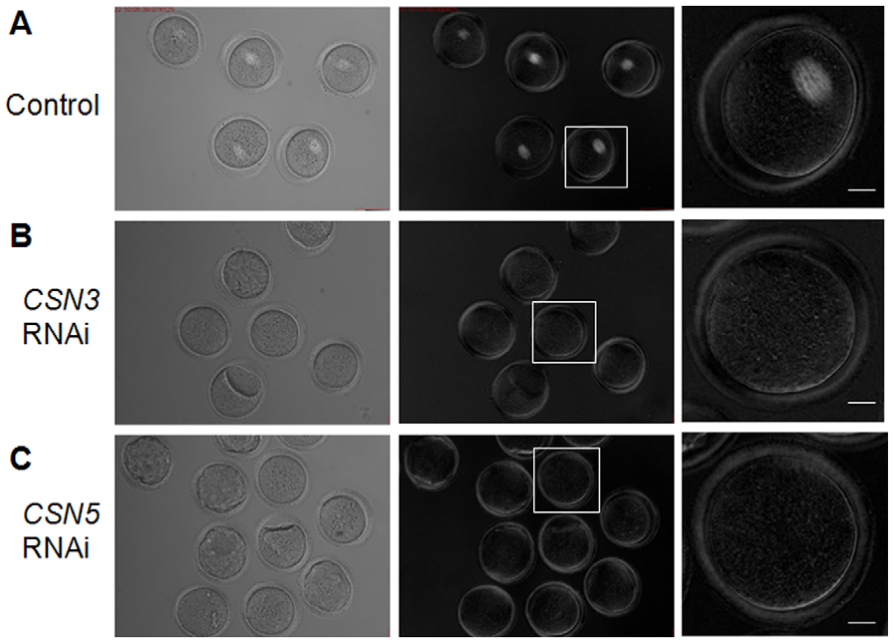
Down-regulation of *CSN3* or *CSN5* caused meiotic spindle disassembly. Spindle structure was observed noninvasively using Polscope microscopy. (A) Control MI oocytes cultured for 8 h showed normal barrel-shaped spindles. (B, C) After RNAi treatment followed by 16 h culture, oocytes were arrested at the MI stage and showed no spindle structure. Left panel, bright field; middle panels, dark field; right panels, magnified view of boxed area from the middle panels. Original magnifications ×200 (Left and middle panels). Bar s = 20 µm.

**Figure 5:**
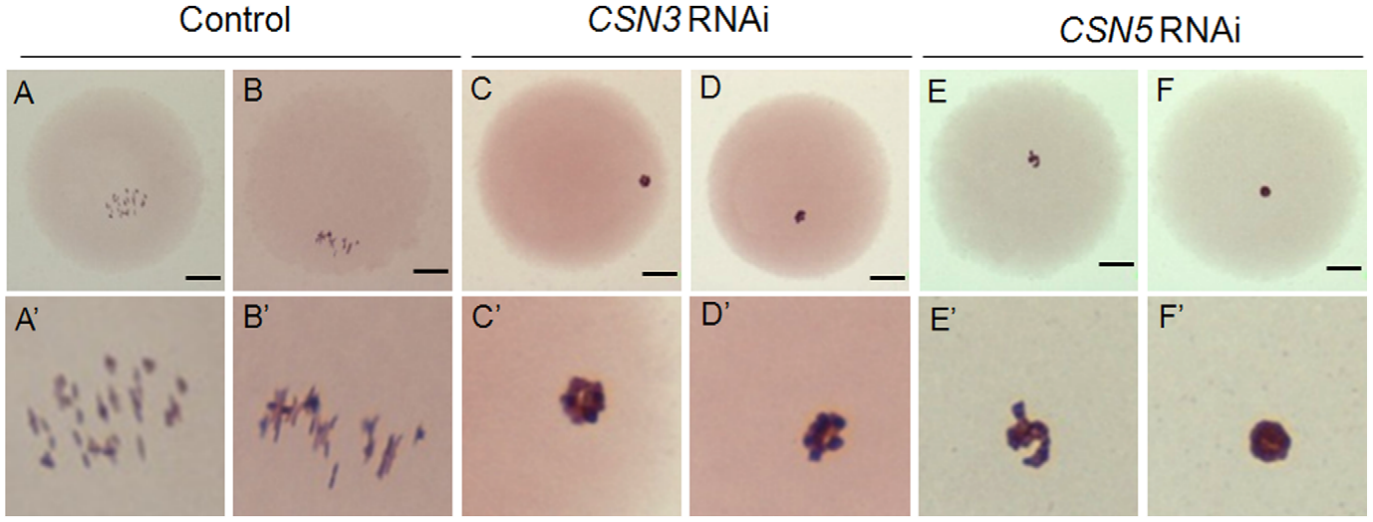
Down-regulation of *CSN3* or *CSN5* caused chromosome aggregation. (A, B) Control MI oocytes showing typical chromosome configuration. (C, D) *CSN3* and (E, F) *CSN5* RNAi-treated oocytes which were arrested at MI showed abnormally aggregated chromosomes. Upper panel (A–F), view of oocytes by optic microscopy (×400); Lower panel (A′–F′), Magnified view of upper panel. These microphotographs were digitally processed to increase magnification. Bars = 25 µm.

**Figure 6:**
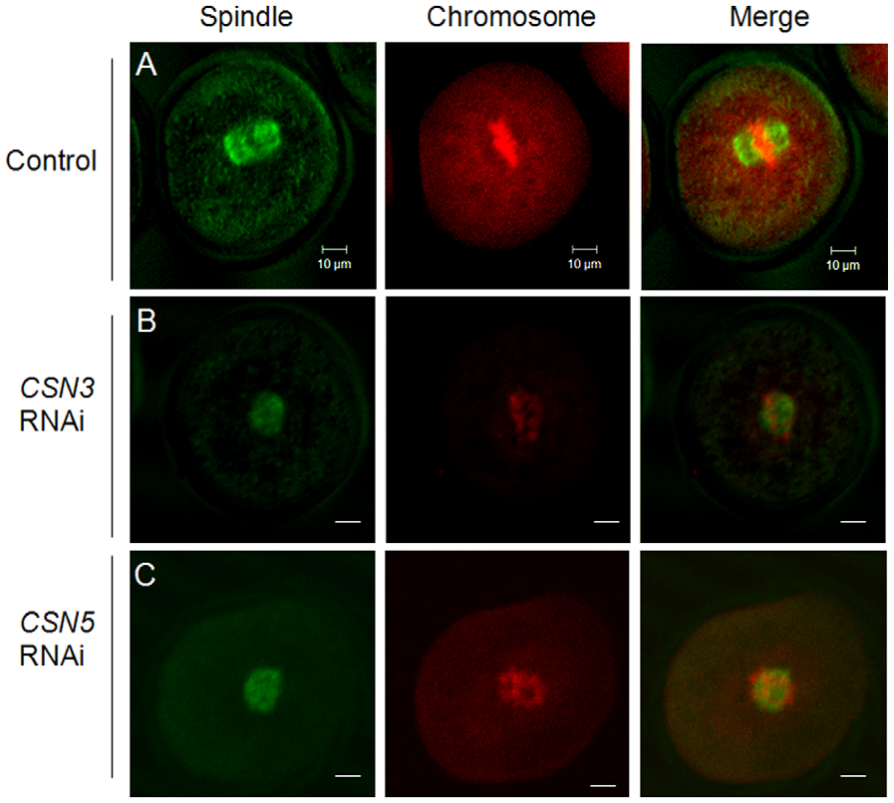
Immunofluorescence staining of the spindle and chromosomes. Spindles were stained with α-Tubulin antibody (green) and chromosomes were counterstained with propidium iodide (red). (A) Control MI oocytes cultured for 8 h. (B, C) RNAi-mediated knockdown of *CSN3* (B) and *CSN5* (C) arrested oocytes at the MI stage and the oocytes showed abnormally aggregated spindle and chromosomes. Left panel, spindle structure; middle panel, chromosome; right panel, merged image. Bar = 10 µm.

Because oocytes failed cytokinesis, we evaluated the meiotic spindle structure noninvasively by Polscope visualization. Control MI oocytes exhibited the clear barrel shape of normal characteristics of meiotic spindles. However, RNAi-treated oocytes showed no spindles under Polscope (Fig. 4). Moreover, control MI oocytes showed well-aligned chromosomes at the metaphase plate, while *CSN3* or *CSN5* RNAi-treated oocytes showed abnormally aggregated chromosomes (Fig. 5C–F). Results of immunofluorescence staining of DNA and the spindle provided further confirmation of these results (Fig. 6). We confirmed that the spindles and chromosomes were not normal and aggregated at the center of the RNAi-treated oocytes (Fig. 6B, C).

### MPF activity was changed as a result of *CSN3* or *CSN5* knockdown

**Figure 7:**
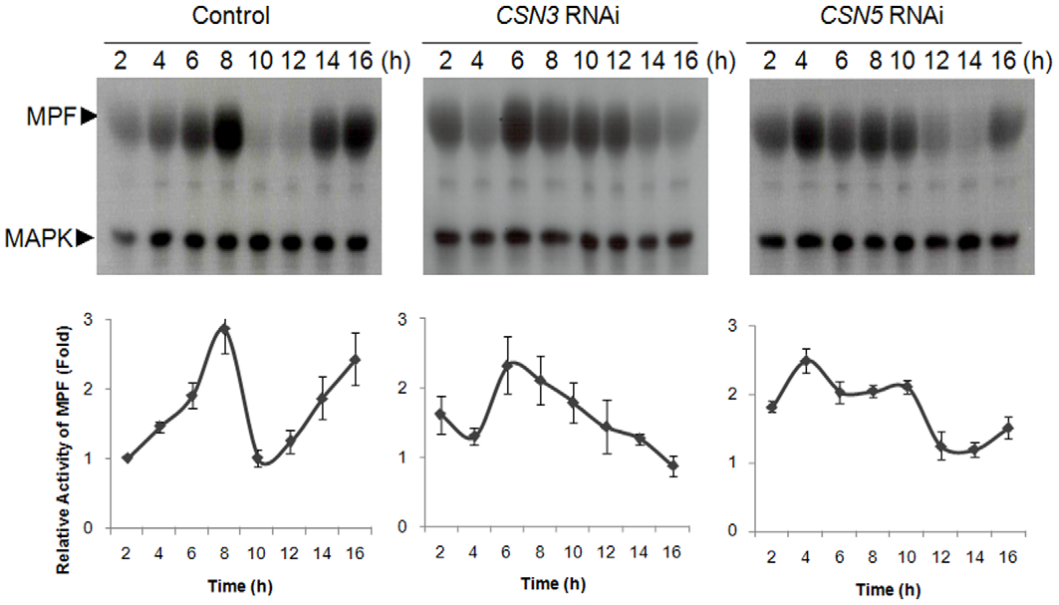
Dual kinase activity assay after *CSN3* or *CSN5* RNAi. MPF and MAPK activities were assessed by measuring the level of phosphorylation of Histone H1, a substrate of MPF, and myelin basic protein, a substrate of MAPK. Oocytes were collected every 2 h during *in vitro* maturation. Lower panel, relative MPF activity was determined by quantifying phosphorylation of substrates and was expressed relative to that of control oocyte at 2 h.

We evaluated the effect of RNAi treatment of *CSN3* or *CSN5* on activities of the two important kinases, MPF and MAPK. In control oocytes, MPF activity increased at 8 h, decreased rapidly, and then rose again during completion of meiosis (Fig. 7). Following *CSN3* and *CSN5* knockdown, changes of MPF activity displayed perverted patterns. Activity of MPF in RNAi-treated oocytes sustained around 8–10 h both in *CSN3* and *CSN5*, and showed no fluctuations of decrease and increase as shown in the control oocytes. However, we cannot explain why CSN3 and CSN5 RNAi showed different pattern of changes in MPF activity at this time. On the other hand, relatively the same activity of MAPK was detected throughout the meiotic cell cycle.

### RNAi-induced abnormal proteolysis of Cyclin B1 and Securin

**Figure 8:**
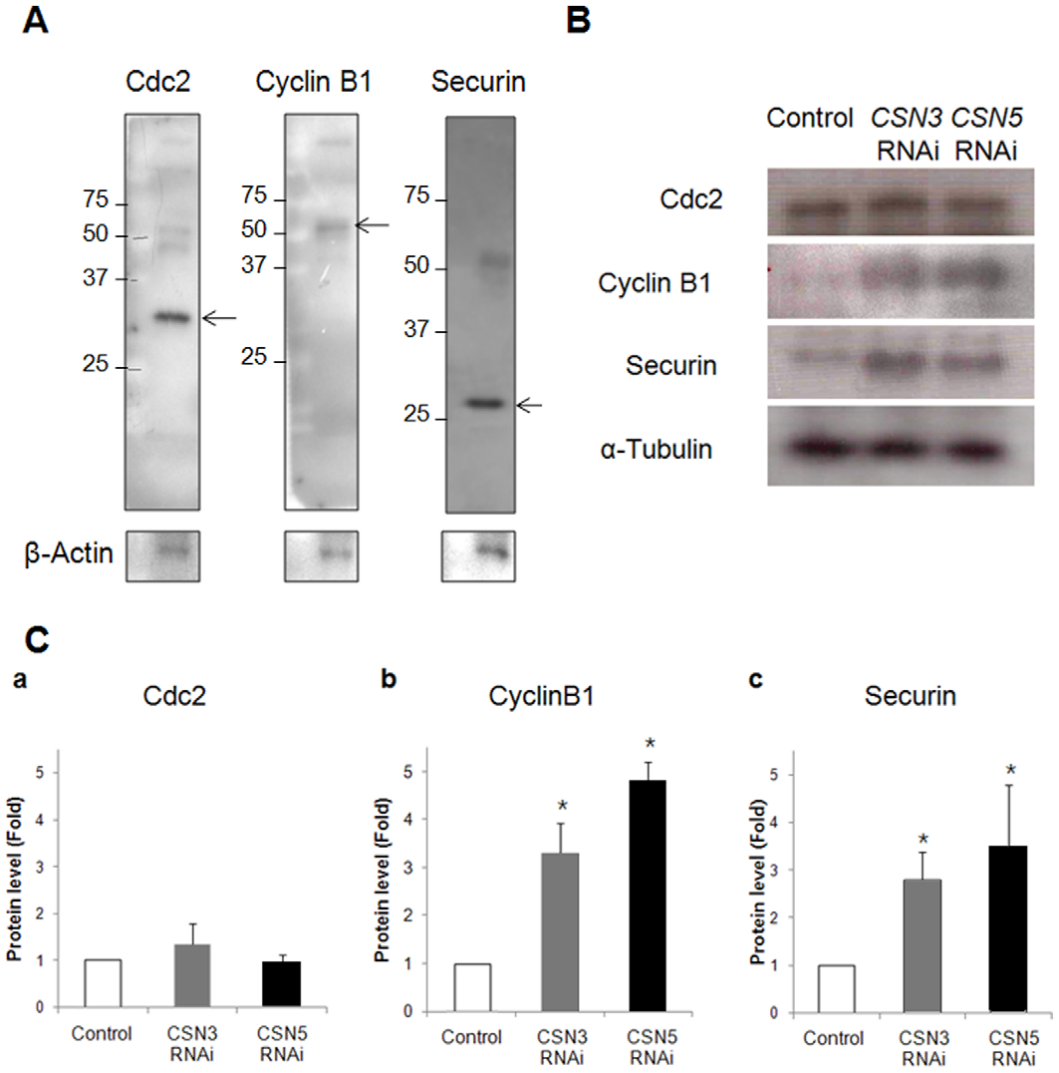
The level of APC/C substrates changed following *CSN3* or *CSN5* knockdown. (A) Western blot analysis of Cdc2, Cyclin B1, and Securin by using 100 oocytes. Numbers on the left side of the band indicate the sizes (kDa) of the protein markers, while arrows indicate the specific proteins. (B) Western blot analysis of Cdc2, Cyclin B1, and Securin by using 100 oocytes. (C) Relative protein level of each band was calculated and presented as the mean ± SEM by measuring the area and data are presented in comparison to appropriate control bands for each protein. Asterisks indicate statistically significant differences compared to control group (p<0.05).

Because MPF inactivation was delayed, we evaluated the degradation of MPF components. It has been reported that the decreased MPF activity after 8 h is due to Cyclin B1 degradation via poly-ubiquitination by the APC/C [30]. Therefore, APC/C activity can be measured indirectly by determining the protein levels of the APC/C substrates, Cyclin B1 and Securin (Fig. 8). Because oocytes cultured for 10 h initiate polar body extrusion despite lower MPF activity, the control oocytes possessed a lower level of Cyclin B1 protein. In RNAi-treated oocytes, the Cyclin B1 level was 3–5-fold higher than the control oocytes, suggesting decreased proteolysis. Likewise, we observed sustained Securin expression that indicating the Securin proteolysis was decreased in RNAi-treated oocytes. It also demonstrated indirectly the lower APC/C activity. However, the level of Cdc2 was unaffected, indicating that the altered MPF activity following *CSN3* and *CSN5* knockdown may have resulted from diminished APC/C activity.

## Discussion

The CSN is a multimeric complex consisting of eight subunits (*CSN1* to *CSN8*) that share significant sequence homology with the eight subunits of the 26S proteasome lid complex [31]. The CSN regulates deneddylation of the SCF (Skp1, Cullins, F-box proteins) E3 ubiquitin-ligase complex, which controls ubiquitin-dependent protein degradation and phosphorylates IkBα, c-Jun, and p53 [32]. Previous studies have reported that CK2 and protein kinase D associate with CSN in human erythrocytes [5] and that phosphoinositide 3-kinase binds to CSN1 [4]. Many reports have demonstrated that mutation of the CSN results in developmental defects, indicating an essential role for the CSN in mammalian cell cycle regulation and early development [21], [23], [24], [33].

Double stranded RNA microinjected into the oocyte cytoplasm cleavages homologous endogenous mRNA, but not the endogenous proteins already synthesized in the cytoplasm. In the present study, mRNA was decreased almost completely and protein was diminished around 40%. This remaining protein might be remnant protein synthesized before microinjection of dsRNA.

Studies have reported that disruption of individual CSN subunits can lead to a failure in CSN complex formation. In particular, *CSN8* is undetectable in *CSN*3-null mouse embryos [24]. Disruption of *CSN2* diminishes *CSN1* and *CSN8* levels [23]. Furthermore, siRNA-mediated knockdown of *CSN8* reduced *CSN* 3, 5 and 7 protein levels, while *CSN* 1, 2, 4, and 6 remained unaffected [34]. When *CSN3* was down-regulated in HeLa cells, CSN5 and CSN8 protein was reduced; however, *CSN5* down-regulation produced no significant effect on the whole complex [35]. Interestingly, in this study, we show that RNA-induced knockdown of *CSN5* reduced only CSN5 itself but did not affect expression of CSN3, whereas down-regulation of *CSN3* reduced the protein level of CSN5 as well as CSN3 itself. These data suggest that *CSN3* and *CSN5* have different positions in the assembly of the whole complex in the oocytes. It also proposes that *CSN3* down-regulation affects the stability of the CSN complex directly, while the complex could still assemble in the absence of CSN5.

There are several conflicting reports regarding the role of *CSN5* on the CSN complex assembly. Down-regulation of this subunit resulted in a stable complex in *Drosophila* [21] but not in *Arabidopsis* [36]. Various small complexes can be formed by a subset of CSN subunits regardless of the presence of *CSN5* [13], [37], [38]. Research has shown that *CSN5* actively participates in important biological functions, both as a part of the CSN holocomplex and on its own [39]. Therefore, it seems likely that, while *CSN3* down-regulation impedes assembly of the whole complex, *CSN5* down-regulation may not affect the other subunits.

During its maturation, an oocyte undergoes two cellular divisions in a sequence of events that are tightly regulated temporally and controlled by the cytoskeleton. Microtubules form the spindle and segregate homologous chromosomes when the bipolar spindle assembles with the chromosomes to form the metaphase plate [39], [40]. In addition to this dynamic spindle structure, the CSN controls microtubule filament stability to modulate cell division. Inactivation of the CSN in *C. elegans* results in defects in spindle positioning and elongation, as well as central positioning of the reforming nuclei [22]. The CSN also regulates mitosis by interacting with end-binding protein 1 [35]. In this study, at our knowledge, we for the first time found that the CSN also regulates the spindle dynamics and meiosis in the mouse oocytes. The molecular regulatory mechanism and relationship between CSN, especially with each subunit, and spindle formation and movement of chromosomes require further studies.

MPF and MAPK are well known two important kinases involved in regulating oocyte maturation [41], [42]. When oocytes are arrested at G2-phase, Cdc25 phosphatase becomes inactive while Wee1 kinase is constitutively activated by cAMP-dependent protein kinase A (PKA). Upon a surge of luteinizing hormone, phosphodiesterase type 3A is activated, inducing a decrease in cAMP levels which inactivates PKA and in turn activates MPF. During meiotic maturation, MPF activity is highest in metaphase I, drops as oocytes exit meiosis I, increases again, and peaks in metaphase of meiosis II [43]. The initial decrease in MPF activity is due to the degradation of Cyclin B1, a component of MPF. This ubiquitin-mediated degradation is triggered by increased levels of active Cdc2 [44]. In mitotic cells, most Cyclin B accumulates in late-G2. However, in mouse oocytes less Cyclin B1 is present [45], [46], suggesting that MPF activity could be regulated tightly. According to the assessment of kinase activity in the absence of CSN subunits in the present study, changes of MPF activity in RNAi-treated oocytes fluctuate different with that of control, indicating dysfunction of the activation/inactivation system for MPF components, Cdc2 and Cyclin B1. Therefore, we hypothesized that sustained MPF activity around 8–10 h may due to decreased APC/C activity, that regulates Cyclin B degradation, by RNAi-mediated knockdown of *CSN3* or *CSN5*.

**Figure 9:**
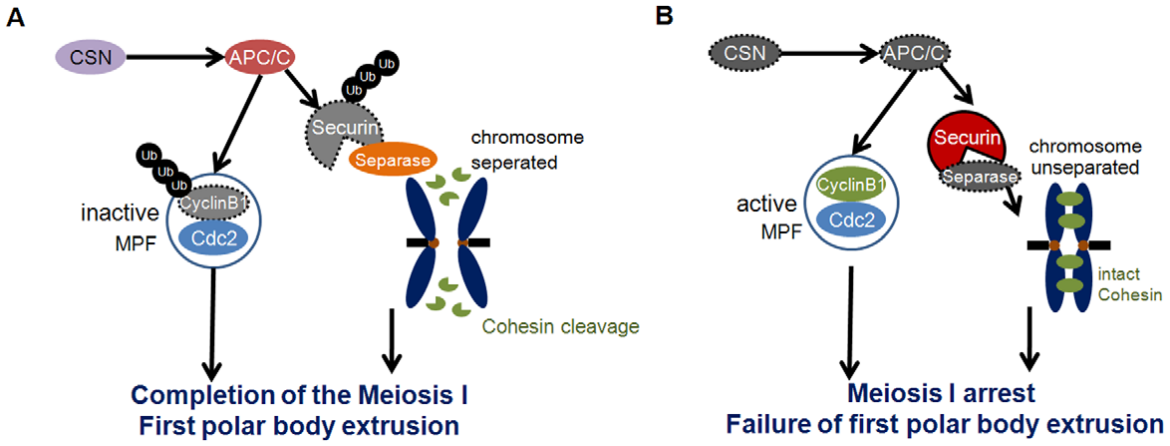
A schematic diagram depicting the proposed role of CSN in the control of meiotic chromosome segregation in oocytes based on loss-of-function of *CSN3* or *CSN5*. (A) CSN regulates APC/C activation, followed by degradation of Cyclin B1 and Securin. Degradation of Cyclin B1 induces MPF inactivation, while degradation of Securin induces activation of Separase that brings Cohesin cleavage and chromosome separation. Following these sequences, meiosis I is completed and first polar body is extruded. (B) When normal CSN is absent, APC/C is not activated, and degradation of Cyclin B1 and Securin are also failed. Maintained MPF activity and intact Securin resulted is maintained high, cohesions are intact, so chromosomes cannot be separated. And then meiosis I is failed; the oocytes are arrested at MI stage.

A cell cycle-regulated ubiquitin ligase, APC/C, is a high molecular weight complex composed of at least 11 subunits that induces ubiquitin-dependent degradation of cell cycle factors [47]. APC/C orchestrates mitosis by controlling anaphase entry and its progression, exit from mitosis, and the G1-phase [47]. During cell division, APC/C initiates chromosome segregation by ubiquitinating Cyclin B and Securin, an inhibitor of the protease Separase. In meiosis, APC/C-mediated degradation of Cyclin B is required for the metaphase-to-anaphase transition [43], moreover, the activity that ubiquitination of Cyclin B1 is involved in exit from MII [48]. It is well established that the CSN interacts physically with the APC/C and regulates several targets including Cyclin A and Cdc6 in human U2OS osteosarcoma cells [49]. When the CSN was down-regulated, SnoN and Cdc6 were increased, while Cyclin A decreased and Cyclin B remained unchanged. Among the substrates, degradation of Cyclin A is related to Cdc20 [50], and SnoN and Cdc6 is related to Cdh1 [51], [52]. Therefore, one could speculate that the regulation of APC/C by the CSN is highly specific to substrate and cell cycle. Our results demonstrate that knockdown of *CSN3* or *CSN5* led to a decreased proteolysis of Cyclin B1 and Securin, suggesting altered activity of APC/C after RNAi treatment (Fig. 9). Furthermore, relatively consistent Cdc2 levels with maintained Cyclin B1 indicating that low APC/C activity blocked MPF inactivation. This failure of APC/C activation and subsequent changes in MPF activity may induce the spindles aggregation and concurrent abnormal chromosomes.

In addition, Securin, another substrate of APC/C, is degraded as Cyclin B1 and thereafter Separase becomes active to break the cohesion up between sister chromatids [53]. Without the CSN, Securin was not degraded during the transition from metaphase to anaphase, and consequently Separase is not activated resulting un-separated and aggregated chromosomes with microtubules.

In conclusion, we report that *CSN3* or *CSN5* in GV oocytes is important in completion of meiotic cell cycle and the deletion of arrested oocytes resulted in abnormally arranged meiotic spindles and chromosomes. Our results may contribute to further study of molecular mechanisms in regulation of meiotic spindle formation and chromosome segregation.
